# The hydrogen sublattice in hydrated molybdenum trioxides: Insight from multi-energy neutron scattering

**DOI:** 10.1063/4.0000785

**Published:** 2025-11-11

**Authors:** Kacper Drużbicki, Erwin Lalik, Robert Kosydar, Matthias Gutmann, Ivan da Silva, Svemir Rudić, Fabio Orlandi, Dmitry Khalyavin, Pascal Manuel, Matthew Krzystyniak

**Affiliations:** 1 Centre of Molecular and Macromolecular Studies, Polish Academy of Sciences, Sienkiewicza 112, 90-363 Lodz, Poland; 2 Jerzy Haber Institute of Catalysis and Surface Chemistry, Polish Academy of Sciences, ul. Niezapominajek 8, 30-239 Cracow, Poland; 3 ISIS Neutron and Muon Source, STFC Rutherford Appleton Laboratory, Harwell OX11 0QX, United Kingdom

## Abstract

Molybdenum oxides have attracted considerable attention in heterogeneous catalysis and energy storage applications owing to the unusual chemical flexibility of the Mo center. Unlike many transition metals, molybdenum can shift between several oxidation states without losing structural integrity, largely due to the stabilizing role of oxo-bridged linkages. This versatility gives rise to an extraordinary diversity of structural motifs that can be tailored for specific catalytic and electrochemical functions. In this study, we investigate the elusive structure and nuclear dynamics of the monohydrate (MoO_3_ · H_2_O) and dihydrate (MoO_3_ · 2H_2_O) phases of β-MoO_3_, an important family of precursors for molybdenum oxide–based hybrid materials. We employ a combined experimental and computational approach to explore the local environment and nuclear dynamics of protons in water confined within the interlamellar space of the β-MoO_3_ layers. High-resolution neutron diffraction confirms the established structure of the dihydrate phase while revealing hydrogen-sublattice disorder in the metastable monohydrate. Complementary computational analysis, including harmonic lattice dynamics and *ab initio* Born–Oppenheimer molecular dynamics simulations, provides deeper insight into proton confinement in these systems, yielding plausible models of their local structure. These findings further validated through temperature-dependent inelastic neutron scattering and neutron Compton scattering, which probe the vibrational response and proton momentum distributions, respectively. The joint analysis of experimental data and molecular dynamics simulations identifies rotationally bound, orientationally disordered water molecules as the mechanism underlying proton disorder in β-MoO_3_ · H_2_O. Overall, the results reveal pronounced differences in water ordering and proton dynamics between the mono- and dihydrate forms, offering a detailed quantum-mechanical description of the hydrogen behavior in hydrated molybdenum trioxides and highlighting the interplay between the thermal effect and the confinement-induced local proton dynamics.

## I. INTRODUCTION

Molybdenum oxides combine rich redox flexibility with structurally robust—yet soft—oxo-bridged frameworks that feature low activation barriers for ion intercalation.^1^ They exhibit remarkable functionality and application potential due to their chemical diversity and highly tunable physical properties.^1–4^ The versatility of molybdenum trioxides in selective oxidation catalysis and electrochemical energy storage arises from the redox flexibility of Mo active centers. This permits modulation of crystal structure, morphology, oxygen vacancies, and dopant distribution, allowing the electronic states to be tuned for a wide range of specific applications.^5–18^

Their polymorphism spans insulating MoO_3_ phases ( α, β, and hexagonal) through metallic MoO_2_ and Magnéli-type suboxides, reflecting the rich connectivity of MoO_6_ octahedra and the ease of oxygen-defect formation across the Mo–O system.^2,7,9,19^ The corresponding hydrates form a layered family, ${\text{MoO}}_{3}\cdot$ H_2_O with x≈0.3–2, which can be topotactically dehydrated along gallery directions to yield the β-phase.^20–24^ These hydrates are not only the precursors to high-surface-area Mo-oxide catalysts and mixed-oxide derivatives but provide controllable galleries and hydrogen-bond networks that modulate transport, making them attractive as electrode hosts where small ions (e.g., Li^+^, ${\text{NH}}_{4}^{+}$) intercalate with comparatively low penalty barriers. Also, in practice, the hydrated phases can outperform anhydrous analogues as electrodes, emphasizing the importance of water content and local bonding in the electrochemical applications.^2,25–30^ For consistency with current crystallographic conventions, we deliberately avoid the ambiguous term molybdic acid and instead refer explicitly to MoO_3_ ·*x*H_2_O phases.^2^ Notably, an important body of information has been provided by mineralogy, complementing studies on synthetic materials.

Within the hydrate family, the dihydrate form, β- ${\text{MoO}}_{3}\cdot 2$ H_2_O is well established crystallographically (space group $P2_{1}/n$), including the exact positions of deuterium atoms from neutron diffraction^31^ and has been earlier found in nature (*Sidwillite*).^32^ The hemihydrate framework and its topotactic links on dehydration are known from thermo-diffractometry.^33^ Both known monohydrates, α- ${\text{MoO}}_{3}\cdot$ H_2_O (*Raydemarkite*; $P\bar{1}$) and β- ${\text{MoO}}_{3}\cdot$ H_2_O (*Virgiluethite*; $P2_{1}/c$), have been recently discovered as natural minerals and studied in detail with single-crystal x-ray crystallography, yet leaving the hydrogen sublattice unresolved.^34^ These natural analogues demonstrate that ${\text{MoO}}_{3}\cdot x$ H_2_O is not merely a family of preparative intermediates but a suite of geologically realized frameworks with robust topotactic links to anhydrous MoO_3_.

Despite this progress, the local environment and nuclear dynamics of intercalated water remain unresolved. Vibrational fingerprints (O-H stretches, bendings, and librations) are exquisitely sensitive to hydrogen bonding, but x-ray and Raman probes have intrinsically low sensitivity to hydrogen. Neutron methods overcome this limitation, enabling otherwise inaccessible insight into the hydrogen sublattice. In the present work, we target the missing information on water structure and dynamics in the β-MoO_3_ · 2H_2_O and β-MoO_3_ · H_2_O. We combine high-resolution neutron powder diffraction (NPD) with inelastic neutron scattering (INS) and neutron Compton scattering (NCS) to probe vibrational spectra and single-particle proton momentum distributions. An extensive experimental protocol was supported by first-principles calculations within the solid-state formulation of density functional theory (DFT).

## II. METHODS

### A. Sample preparation

Powder samples of β-MoO_3_ · 2H_2_O and β-MoO_3_ · H_2_O were synthesized according to the method of Freedman.^35^ Sodium molybdate dihydrate (Na_2_ ${\text{MoO}}_{4}\cdot$ 2H_2_O, Sigma Aldrich) was dissolved in distilled water at room temperature. Subsequently, 5 M HNO_3_ (POCH) was added, and the solution was stirred for several hours. The mixture was then kept in a closed vessel for 1 week, with occasional stirring. After 8 days, the vessel was opened and left undisturbed. Once the first crystallites appeared, further precipitation was observed as the solvent gradually evaporated. After two weeks, the yellow solid was collected by gravity filtration, washed carefully with 4 M HNO_3_ until the filtrate was colorless, then washed with a large excess of distilled water, and dried in air at room temperature. The resulting yellow powder was identified as β- ${\text{MoO}}_{3}\cdot$ 2H_2_O [see Fig. S1(a)]. Subsequently, part of the product was heated in air at 70 °C for 3 h. The material changed the color from yellow to orange [Fig. S1(b)], with a ∼ 10% weight loss corresponding to dehydration, yielding β- ${\text{MoO}}_{3}\cdot$ H_2_O. The quality of the materials was verified by laboratory powder x-ray diffraction (PXRD; see Fig. S2), revealing patterns consistent with those reported by Günter.^20^

Following materials characterization, the samples were subjected to a multi-energy neutron scattering campaign at both target stations I and II of the ISIS Pulsed Neutron and Muon Source (Oxfordshire, United Kingdom). Data reduction and processing were performed using the Mantid software package.^36^

### B. Neutron diffraction

The ambient-temperature NPD data for β-MoO_3_ · 2H_2_O and β-MoO_3_ · H_2_O specimens were collected on the high-resolution cold-neutron powder diffractometer WISH installed at ISIS target station II.^37^ Data were grouped into nominal diffraction angles and subjected to Le Bail analysis,^38^ using the JANA2020 program.^39^ The total-profile refinement using pseudo-Voigt peak profiles was performed jointly for the three selected diffraction banks. For the dihydrate β- ${\text{MoO}}_{3}\cdot$ 2H_2_O, the results are fully consistent with the structural model resolved by Crouch and Baker,^31^ revealing a distorted, yet well-defined water sublattice. In contrast, the monohydrate β- ${\text{MoO}}_{3}\cdot$ H_2_O exhibits broadened reflections, in agreement with the results reported by Boudjada and coworkers on D1B ( λ=2.52 Å).^40^ While our present data recorded on WISH offers a superior resolution, the trial Rietveld refinements indicate smeared proton positions, consistent with positional and/or orientational disorder of the water molecules. Therefore, we restricted the analysis to Le Bail refinement, using it as a basis for model selection among the plausible structural candidates proposed by *ab initio* calculations described below.

### C. Inelastic neutron scattering

The INS spectra for both specimens were recorded using a TOSCA spectrometer,^41–47^ located at ISIS target station I. The powdered samples were loaded into a rectangular aluminum sample cell, sealed with indium wire, and cooled to the base temperature. Following the base-temperature measurements, spectra were collected over the temperature range of 25–300 K.

### D. Neutron Compton scattering

The NCS spectra for both hydrated molybdenum trioxide samples were recorded at the VESUVIO thermal-to-epithermal neutron station,^48–50^ located at ISIS Target Station I. To preserve the structural integrity of both samples, the specimens previously used on TOSCA were subsequently used on VESUVIO. The NCS spectra were measured at 50, 100, 200, and 300 K, with the temperature stabilized using a sample environment consisting of a closed-cycle refrigerator (CCR).

There exists an extensive body of literature on the subject of NCS data reduction and analysis, and the reader is referred to it for a detailed insight.^48–56^ For the sake of brevity, here, we discuss only the main aspects of the data reduction and analysis scheme that are relevant to the present work.

Prior to the NCS experiments with the β-MoO_3_ · H_2_O and β-MoO_3_ · 2H_2_O specimens, the NCS spectra of an empty instrument—with only the CCR sample environment placed inside the instrument and facing the incoming neutron beam—were recorded. All spectra were recorded in the time-of-flight (TOF) domain. The empty-instrument TOF spectra were then subtracted from the TOF spectra recorded on VESUVIO for both specimens in order to remove the unresolved background.

The raw, background-corrected TOF spectra of both specimens were subjected to an iterative numerical procedure whose logic is dictated by the impulsive and fully incoherent nature of NCS scattering.^48–56^

The Compton scattering process proceeds in a “one-atom-at-a-time” manner, and each NCS spectrum consists of a series of recoil peaks centered at distinct values of TOF, at which the conservation of energy and momentum of the target–projectile system is fulfilled for stationary target nuclei. The recoil peaks are Doppler-broadened in the TOF domain by the nuclear momentum distributions (NMDs) of the target nuclei.

The kinematics of the impulsive NCS process dictate that there is no Compton backscattering of neutrons from protons. Thus, for any compound under investigation, the VESUVIO backscattering neutron detectors record spectra consisting of recoil peaks of all isotopic species apart from hydrogen, while the forward VESUVIO detectors record NCS spectra of all isotopic species.

However, the resolution in the TOF domain with which the recoil peaks of isotopes heavier than protons are recorded is higher in backscattering than in forward scattering. Thus, the fitting parameters characterizing the shapes of the recoil peaks of heavier isotopes can first be fitted in backscattering and then fixed when fitting the forward-scattering NCS spectra, which are analyzed solely to fit the shapes of the proton recoil peaks accurately.

For the analysis of the shapes of the recoil peaks, the underlying NMDs of isotopes heavier than protons were modeled as purely isotropic Gaussians, with only the width (the standard deviation) of the underlying NMD free in fitting, and the relative integral intensity of each recoil peak fixed by the known compound formula unit and the values of the total bound scattering cross sections of all isotopic species in the sample. In the case of the hydrogen recoil peaks, two models—an isotropic univariate Gaussian and an anisotropic bivariate Gaussian—for the underlying NMD were compared using a Markov Chain Monte Carlo (MCMC) approach, as implemented in the Fabada algorithm,^57^ integrated in the Mantid computational environment.^36^

Because high-incident-energy epithermal neutrons are used on VESUVIO for Compton scattering, there is a finite probability of multiple scattering in the sample and the aluminum container. Moreover, because epithermal neutrons scattered from protons are recorded on VESUVIO by gamma-ray–sensitive detectors—as opposed to the backscattering detectors that record neutrons directly—raw NCS data correction procedures must be applied to account for multiple scattering (MS) and sample-composition–dependent gamma background (GB). These two types of corrections are applied to the raw NCS data in the TOF domain within an iterative, self-consistent numerical procedure consisting of the following steps:

The procedure starts by treating the backscattering data, which is analyzed sequentially on a detector-by-detector spectrum basis:

1. The raw spectra are fitted with a collection of Gaussian NMDs, and the initial guess of the MS correction is obtained.
2. The initial MS contribution is subtracted from the raw data.
3. The corrected spectra are refitted, and the next-iteration estimate of the MS correction is calculated.

The sequence consisting of steps 1–3 is repeated multiple times until convergence is achieved. For the samples under investigation, two iterations of the above-mentioned procedure were sufficient.

Following completion of the backscattering data reduction procedure, the set of converged NMD peak widths of isotopes heavier than protons is used to fix the shape of the non-proton part of the NCS spectra recorded by the forward-scattering detectors within the following iterative procedure, which is applied sequentially on a detector-by-detector spectrum basis.

1. The raw spectra are fitted with a collection of Gaussian NMDs, and the initial estimates of the MS and GB backgrounds are obtained.
2. The initial MS and GB corrections are subtracted from the raw data.
3. The corrected spectra are refitted, and the next-iteration estimates of the MS and GB corrections are calculated.

Following completion of the second part of the NCS data treatment procedure, the final third part consists of fitting the recoil peak of hydrogen only and involves the following steps:

1. The fitted recoil peaks of masses other than protons are subtracted from the total spectra, and the recoil peaks of hydrogen are isolated in the TOF domain.
2. The isolated recoil peaks of hydrogen are transformed (focused) into the longitudinal momentum domain of hydrogen to form the experimental proton momentum distribution.
3. The experimental proton momentum distribution is fitted with multiple models (in this case, univariate and bivariate Gaussians), and the probability of each model describing the data is ranked against the others using the MCMC algorithm.

### E. *Ab initio* modeling

Theoretical calculations in solid-state were carried out under periodic boundary conditions (PBCs) within the plane wave pseudopotential formalism, as implemented in the *Cambridge Serial Total Energy Package* (Castep, version 21.11).^58,59^ The calculations comprised several stages, beginning with the construction of plausible models for both the monohydrate and dihydrate forms of β- ${\text{MoO}}_{3}\cdot x$ H_2_O, followed by electronic structure calculations and accurate geometry optimization. Subsequently, the phonon band structure was determined to assess mechanical stability, and finally, finite-temperature *ab initio* molecular dynamics (AIMD) simulations were performed.

For the dihydrate phase, we relied on the room-temperature crystallographic model of perdeuterated β- ${\text{MoO}}_{3}\cdot 2$ D_2_O originally determined by Crouch and Baker (NPD, D1A high-resolution diffractometer at the high flux reactor, ILL, Grenoble, λ = 1.909 Å).^31^ For the monohydrate phase, plausible structural models were primarily constructed based on the recently reported structure of *Virgilluethite*.^24^

For the electronic structure calculations, the same numerical settings were adopted as in our previous work on ${\text{WO}}_{3}\cdot$ H_2_O.^60^ The exchange–correlation functional was treated within the generalized gradient approximation (GGA) using the Perdew–Burke–Ernzerhof (PBE) form, supplemented by the Tkatchenko–Scheffler (TS) semi-empirical dispersion correction.^61,62^ Core states were represented by hard norm-conserving pseudopotentials generated on-the-fly, and the valence wave functions were expanded in a plane wave basis set with a kinetic energy cutoff of 900 eV. To enhance numerical precision, real-space integration over the fast Fourier transform (FFT) grid was carried out using a double-grid method for the evaluation of the charge density and exchange-correlation terms. The maximum reciprocal lattice vector of the FFT grid, $G_{\text{max}}$, was chosen as three-quarters of the ideal grid size, and the fine-grid scaling factor was fixed at 4.^63^ Brillouin-zone integrations were performed using the Monkhorst–Pack (MP) grid adjusted to maintain a constant spacing in reciprocal space. The MP-grid of 0.05 Å^–1^ along with the self-consistent-field (SCF) energy converged with a tolerance of 1 × 10^−12 ^eV per atom was employed throughout this work. The Pulay mixing scheme with a fixed charge occupancy was employed to accelerate convergence of the SCF cycles.

Variable-cell relaxations were carried out at 1 atm using the limited-memory Broyden–Fletcher–Goldfarb–Shanno (LBFGS) algorithm together with a three-point finite basis set correction to reduce the Pulay stresses. All structures were fully optimized to minimize the residual atomic forces, a necessary step for obtaining a well-converged phonon band structure.^63,64^ The convergence thresholds for the Hellmann–Feynman forces and external stress were set to 1 × 10^−5^ eV/Å and 1 × 10^−4^ GPa, respectively.

Following geometry optimization, the vibrational response was examined using harmonic lattice dynamics (HLD) and AIMD simulations. Within the HLD approach, phonon dispersions across the first Brillouin zone (FBZ) were calculated using density functional perturbation theory (DFPT) in the reciprocal-space formalism.^65–68^ The dynamical matrices were evaluated at T=0 K for the primitive cell models, yielding phonon frequencies and eigenvectors used to assess their dynamical stability. Imaginary branches, when present, were further explored via the frozen-phonon method, where atomic displacements along the corresponding eigenvectors were imposed to map the potential-energy surface (PES) and identify the symmetry-reduced configurations, commensurate with a given wave vector.^69^ The HLD results also provided the input for simulations of neutron scattering observables, including INS and NCS. Phonon eigenvalues and eigenvectors were employed for direct simulation of the TOSCA spectrum,^70^ while atom-projected vibrational densities of states (apVDOSs) were used to calculate the NCS observables according to previously established procedures.^52–54^ Analysis of the vibrational modes was carried out using PDIelec,^71^ and INS spectra were simulated with oCLIMAX.^70^

A series of classical AIMD simulations within the Born–Oppenheimer framework (BOMD) was carried out at T = 50, 100, 200, and 300 K for the most plausible structural candidates of both hydrated phases, using the 1×1×1 ( β- ${\text{MoO}}_{3}\cdot 2$ H_2_O) and 2×1×2 ( β- ${\text{MoO}}_{3}\cdot$ H_2_O) simulation cells, respectively. The basis-set quality and numerical settings were identical to those described above, except that the SCF tolerance was relaxed to $5\times 10^{-7}$ eV/atom and the fine-grid multiplier was reduced to the FFT size. Equations of motion were integrated with a 0.5 fs time step. Each AIMD simulation consisted of three stages. First, the initial configurations were equilibrated for approximately 5000 steps in the isothermal–isobaric (NPT) ensemble at ambient pressure. Initially, a Nosé–Hoover thermostat combined with the anisotropic Parrinello–Rahman barostat was applied to both β- ${\text{MoO}}_{3}\cdot 2$ H_2_O and β- ${\text{MoO}}_{3}\;\cdot$ H_2_O. For the monohydrate phase, however, the weaker interlayer bonding made the Parrinello–Rahman scheme prone to fluctuations. Therefore, after equilibrating at 300 K, the subsequent temperature-variable simulations were carried out using an isotropic Andersen barostat to ensure stability. In the second stage, the systems were confined to the average simulation cell derived from the NPT trajectory and propagated further in the canonical (NVT) ensemble. Extended Lagrangian Born–Oppenheimer MD (XL-BOMD) was employed to propagate the electronic wave functions together with the ionic positions. The first 2.5 ps of the NVT run was discarded as equilibration, followed by production runs of 20k and 15k steps for the β- ${\text{MoO}}_{3}\cdot 2$ H_2_O and β– ${\text{MoO}}_{3}\;\cdot$ H_2_O models, respectively.

Based on the apVDOSes obtained from the *ab initio* simulations, the kinetic energy tensors and NMD widths were computed. Throughout all simulations, the system of physical units was adopted, in which the momentum transfer, Q, and the widths of nuclear momentum distributions, σ, are expressed in ${Å}^{-1}$, the elements of kinetic energy tensor, E, as well as the vibrational energy, ω, are expressed in meV, and the nuclear masses in atomic mass units (amu). Consequently, the reduced Planck constant, ℏ, is expressed in units of (meV amu) ${}^{1/2}$ Å, with $\hbar^{2}=4.18$ meV amu Å^2^.^48–50^ All kinetic energy tensors were initially expressed in the orthogonal Cartesian coordinate system.

For atomic species of type *j*, the (k,l) th element ( k,l∈{x,y,z}) of its kinetic energy tensor, $E_{k,l}^{j}$, can be obtained using the following formula:

$$E_{k,l}^{j}=\frac{\int_{0}^{\infty }\frac{\omega }{4}\;g_{k,l}^{j}(\omega )\;\text{coth}\left(\frac{\omega }{2k_{B}T}\right)\;d\omega }{\int_{0}^{\infty }g_{k,l}^{j}(\omega )\;d\omega }$$ (1)

with:^72^

$$g_{k,l}^{j}(\omega )=\int_{0}^{\infty }C_{k,l}^{j}(t)\;\text{exp}\left(-\frac{i\omega t}{\hbar }\right)\;dt,$$ (2)

where $g_{k,l}^{j}(\omega )$ is the Fourier transform of the intra-atomic cross correlation function, $C_{k,l}^{j}(t)$, describing correlations between different Cartesian velocity components:^72^

$$C_{k,l}^{j}(t)=\frac{\left\langle v_{k}^{j}(0)v_{l}^{j}(t)\right\rangle }{\sqrt{\left\langle v_{k}^{j}(0)v_{k}^{j}(0)\rangle \langle v_{l}^{j}(0)v_{l}^{j}(0)\right\rangle }}.$$ (3)

For k=l, $\sum_{k=1}^{3}C_{k,k}^{j}(t)$ reduces to the intra-atomic velocity autocorrelation function for the atom of type *j* and $\sum_{k=1}^{3}g_{k,k}^{j}(\omega )$ to the *j*th atom-type-projected vibrational density of states.

To streamline and unify the computational workflow, we based it on the $g_{k,l}^{j}(\omega )$ functions, for both the HLD and AIMD simulations. To accomplish this, in the case of the AIMD simulations, the $g_{k,l}^{j}(\omega )$ functions were obtained by processing the molecular dynamics trajectories directly in Travis. In the case of the HLD, the $g_{k,l}^{j}(\omega )$ functions were obtained from the HLD phonon outputs using in-house built Matlab scripts inspired by our previous work.^73^

It is important to note that, since the NCS technique is generally assumed not to be site-selective, the kinetic energy tensors, $E=E_{k,l}^{j}$, were obtained in the simulations as site-averaged quantities, for each constituent atomic species. To this end, we employed Travis to compare the numerical values of $E=E_{k,l}^{j}$ obtained from two computational routes. In the first route, the site-averaged E tensors were computed using $g_{k,l}^{j}(\omega )$ summed over all sites. In the second route, individual E tensors were obtained using $g_{k,l}^{j}(\omega )$ calculated independently for each site, and then the tensors were averaged over sites. The site-averaged E tensors obtained in both routes were equal within the numerical precision of the computational scheme.

After computing all six independent matrix elements of the symmetric kinetic energy tensor for each nucleus of mass *M*, the momentum covariance tensor, $\Sigma_{p}$, was obtained using the following relation:

$$\Sigma_{p}=\frac{2M}{\hbar^{2}}\;E.$$ (4)

The symmetric $\Sigma_{p}$ was diagonalized to obtain principal standard deviations, $\left(\sigma_{1},\sigma_{2},\sigma_{3}\right)$ and eigenvectors.

In the next step of the computational protocol, a multivariate Gaussian radial momentum distribution was assumed as follows:

$$n(p)=\frac{1}{{\left(2\pi \right)}^{3/2}|\Sigma_{p}{|}^{1/2}}\text{exp}\left[-\frac{1}{2}\;p^{T}\Sigma_{p}^{-1}p\right].$$ (5)

This radial momentum distribution underlies the longitudinal momentum distribution, which is directly observable in NCS experiments, and relates to its radial counterpart by the Radon transform of the following form:^48–50^

$$J(y,\hat{q})=\int n(p)\;\delta \left(y-p\cdot \hat{q}\right)d^{3}p,$$ (6)

where $\hat{q}$ denotes the unit vector along the momentum-transfer direction and *y* is the longitudinal momentum component along that axis. Equation (6) expresses $J(y,\hat{q})$ as the projection of n(p) onto planes perpendicular to $\hat{q}$.

In the case where the Radon transform (6) is performed along a single direction in space, denoted by $\hat{q}$, and the n(p) is a multivariate Gaussian, the Radon transform yields again a Gaussian longitudinal distribution,

$$J(y,\hat{q})=\frac{1}{\sqrt{2\pi }\;\sigma_{\hat{q}}}\text{exp}\left[-\frac{y^{2}}{2\sigma_{\hat{q}}^{2}}\right],\;\sigma_{\hat{q}}^{2}={\hat{q}}^{T}\Sigma \;\hat{q},$$ (7)

whose variance $\sigma_{\hat{q}}^{2}$ is the projection of the full covariance tensor Σ along the direction of $\hat{q}$.

As the molybdic acid samples were measured in solid powder form, the powder (spherical) averages of $J(y,\hat{q})$ were computed in the next step. For that, $\sigma_{\hat{q}}$ was replaced by its angular average S(θ,ϕ) over all orientations:^48–50^

$${\left\langle J_{\hat{n}}(y)\right\rangle }_{\hat{n}}=N\int_{0}^{\pi }\;\text{sin}\;\theta \;d\theta \int_{0}^{\pi /2}S^{2}(\theta ,\phi )\;e^{-y^{2}/[2S^{2}(\theta ,\phi )]}\;d\phi ,$$ (8)

where

$$N=\frac{1}{\sqrt{2\pi }\;\sigma_{1}\sigma_{2}\sigma_{3}}$$ (9)

and

$$S^{-2}(\theta ,\phi )=\frac{\;{\text{sin}}^{2}\theta \;{\text{cos}}^{2}\phi }{\sigma_{1}^{2}}+\frac{\;{\text{sin}}^{2}\theta \;{\text{sin}}^{2}\phi }{\sigma_{2}^{2}}+\frac{\;{\text{cos}}^{2}\theta }{\sigma_{3}^{2}},$$ (10)

and $\left(\sigma_{1},\sigma_{2},\sigma_{3}\right)$ are the principal standard deviations of n(p) while $\left(\frac{{\left(\hbar \sigma_{1}\right)}^{2}}{2M},\frac{{\left(\hbar \sigma_{2}\right)}^{2}}{2M},\frac{{\left(\hbar \sigma_{3}\right)}^{2}}{2M}\right)$ are the eigenvalues of E. It is important to note that, in general, the kinetic energy tensors are not diagonal in the Cartesian coordinate system, neither for individual sites nor as site-averaged quantities. For that reason, $\left(\sigma_{1},\sigma_{2},\sigma_{3}\right)$ are, in general, not the same as $\left(\sigma_{x},\sigma_{y},\sigma_{z}\right)$.

In principle, two conceptually distinct but mathematically equivalent routes can be followed to obtain the powder-averaged longitudinal momentum distribution ${\left\langle J_{\hat{n}}(y)\right\rangle }_{\hat{n}}$:

(A) *Powder-average the 3D momentum density* n(p) first, by integrating over all orientations of the principal axes of $\Sigma_{p}$, and then compute its Radon transform;
(B) *Compute the directional* $J_{\hat{n}}(y)$ *for each orientation* of n(p) and then average the resulting $J_{\hat{n}}(y)$ over the sphere of directions $\hat{n}$.

For a multivariate Gaussian momentum density, the two operations commute because both the spherical averaging and the Radon transform are linear operators that depend only on rotational invariants of $\Sigma_{p}$. Therefore,

$${\left\langle J_{\hat{n}}(y)\right\rangle }_{\hat{n}}=J\left(y;\;{\left\langle R\Sigma_{p}R^{T}\right\rangle }_{R}\right),$$ (11)

where *R* represent the rotation matrices composed of eigenvectors of $\Sigma_{p}$. Physically, this means that while the powder-averaged tensor $\langle R\Sigma_{p}R^{T}\rangle =(\text{Tr}\;\Sigma_{p}/3)\;I$ (where I is the unit matrix) is isotropic, the resulting ${\left\langle J_{\hat{n}}(y)\right\rangle }_{\hat{n}}$ *still retains information about the distribution of anisotropies* through the directional variance $S^{2}(\theta ,\phi )$ that enters the integral. Hence, anisotropy manifests in the shape of ${\left\langle J_{\hat{n}}(y)\right\rangle }_{\hat{n}}$ even though all orientations are averaged out.

In the simulations, the analytic evaluation route was chosen. It is stable and efficient across extreme anisotropies. In contrast, volumetric and Radon transform-based approaches require adaptive voxel sizes and large domains to capture very small nuclear momentum distribution width values, and the subsequent averaging is sensitive to binning and interpolation. The analytic route provides smooth, normalized ${\left\langle J_{\hat{n}}(y)\right\rangle }_{\hat{n}}$ with modest angular quadrature.

It is worth noting that, for single-crystal and/or oriented samples, in principle, Eqs. (8) and (10) can be used to fit the entire kinetic energy tensors (including off diagonal terms) to experimental data expressed in the longitudinal nuclear momentum distribution domain and then compare the fitted tensors to their counterparts obtained from *ab initio* predictions. However, even in that case, the site averaging of the kinetic energy tensor over inequivalent sites for the same atomic species renders such a fitting procedure very sensitive to noise and experimental data error. In the case of powder samples, the fitting is even more challenging, and, in general, the combined effect of site and powder averaging renders the procedure ill-posed. However, as mentioned above, the powder averaging retains information about the distribution of anisotropies through the directional variance in the Radon transform, and one can retrieve, through fitting, the diagonal elements of the site-averaged kinetic energy tensors. Such an attempt was made in the case of the solid powder molybdic acid data. To this end, numerical experiments were performed, whereby NMDs described by Eq. (8) were fitted to synthetic (numerically powder-averaged) momentum distributions. In each case, the values of $\left(\sigma_{1},\sigma_{2},\sigma_{3}\right)$ that constituted input to the simulations were recovered through fits with satisfactory accuracy.

In the final step of the simulations, the spherically averaged values of the kinetic energies were obtained from the diagonal elements of the simulated kinetic energy tensors as follows:

$$E_{\text{iso}}^{j}=\frac{E_{1,1}^{j}+E_{2,2}^{j}+E_{3,3}^{j}}{3}.$$ (12)

Using the $E_{1,1}^{j}$, $E_{2,2}^{j}$, and $E_{3,3}^{j}$ values, the theoretical predictions for the NMD widths of the multivariate Gaussian longitudinal momentum distributions, $\sigma_{1}^{j},\sigma_{2}^{j},\sigma_{3}^{j}$, were then calculated according to

$$E_{1,1}^{j}=\frac{{\left(\hbar \sigma_{1}^{j}\right)}^{2}}{2M},\;E_{2,2}^{j}=\frac{{\left(\hbar \sigma_{2}^{j}\right)}^{2}}{2M},\;E_{3,3}^{j}=\frac{{\left(\hbar \sigma_{3}^{j}\right)}^{2}}{2M}.$$ (13)

From the simulated NMD widths of the multivariate Gaussian longitudinal momentum distributions, the perpendicular and longitudinal components of bi-variate Gaussian longitudinal momentum distributions, $\sigma_{∥}^{j}$ and $\sigma_{⊥}^{j}$, were then calculated for the comparison with the results of the fitting of the bi-variate Gaussian longitudinal momentum distribution function to the experimental data, according to

$$\sigma_{∥}^{j}=\sqrt{\frac{{\left(\sigma_{1}^{j}\right)}^{2}+{\left(\sigma_{2}^{j}\right)}^{2}}{2}},$$ (14)

$$\sigma_{⊥}^{j}=\sigma_{3}^{j}.$$ (15)

The isotropic NMD widths, $\sigma_{\text{iso}}^{j}$, were then calculated as

$$\sigma_{\text{iso}}^{j}=\sqrt{\frac{{\left(\sigma_{1}^{j}\right)}^{2}+{\left(\sigma_{2}^{j}\right)}^{2}+{\left(\sigma_{3}^{j}\right)}^{2}}{3}}.$$ (16)

In addition to the NCS, INS spectra were simulated from the AIMD trajectories using a recently developed scheme that incorporates an approximate treatment of the Debye–Waller factor, overtone and combination bands, as well as both site and powder averaging and the instrumental resolution of the TOSCA spectrometer.^74^

## III. RESULTS AND DISCUSSION

### A. Structure

**FIG. 1.**
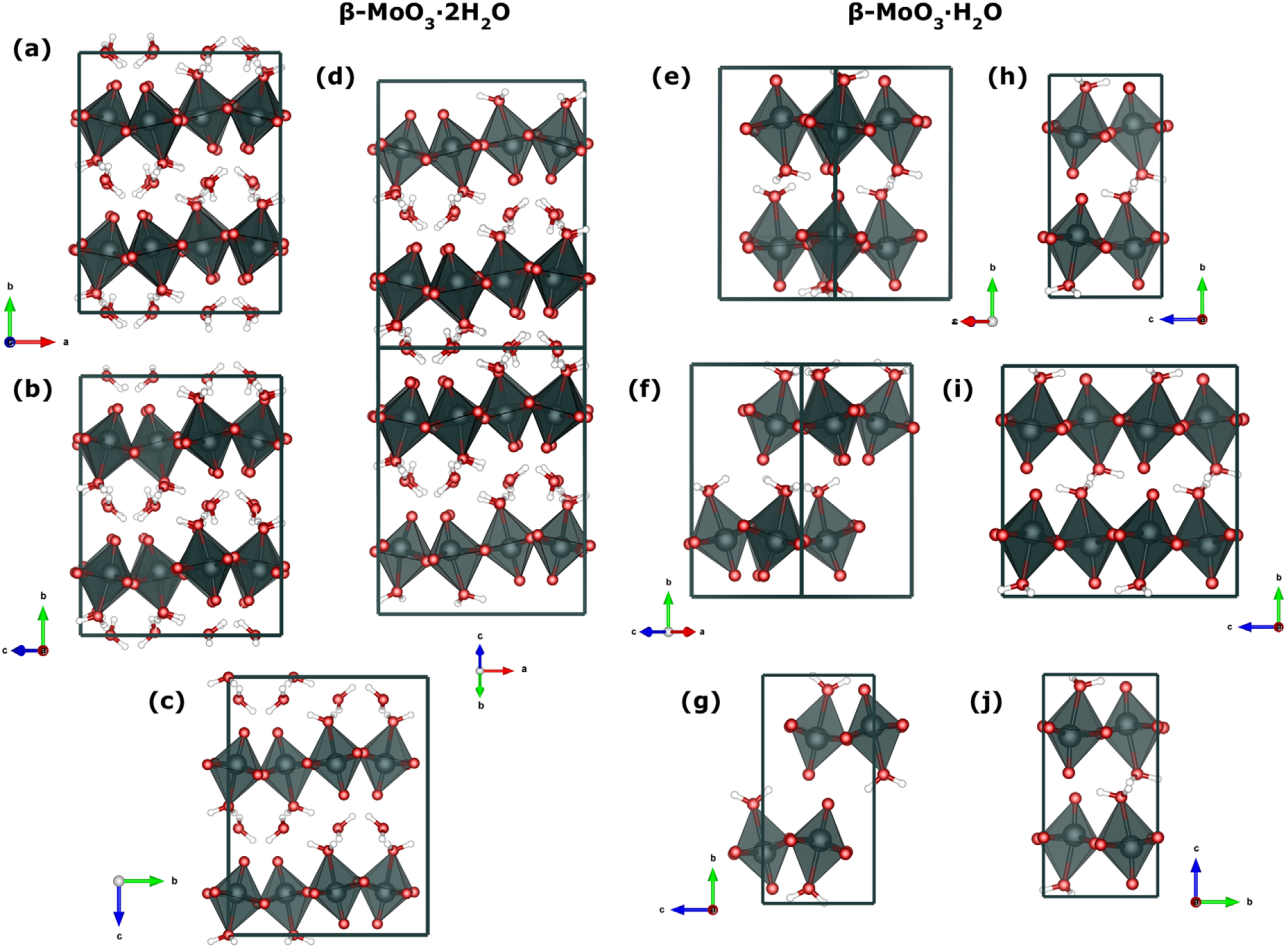
Plausible structural models for hydrated β-MoO_3_. Panels (a)–(d) display dihydrate β- ${\text{MoO}}_{3}\cdot 2$ H_2_O candidates derived from the crystal structure ( ${\text{MoO}}_{3}\cdot 2$ D_2_O, $P2_{1}/n$). Panels (e)–(j): monohydrate β- ${\text{MoO}}_{3}\cdot$ H_2_O variants derived from the *Virgilluethite* topology, differing in water-site occupancy/orientation and the hydrogen bonding motifs (see the main text for more details). All models were screened with HLD as presented in Figs. S3–S4 and deposited in the supplementary material.

As summarized in Fig. 1, we evaluate ten candidate structures (a)–(j) that span the two hydrated β–MoO_3_ compositions, which were optimized using dispersion-corrected plane wave DFT and subjected to numerically accurate HLD calculations. Models (a)–(d) represent the dihydrate β– ${\text{MoO}}_{3}\cdot 2$ H_2_O phase, while the β– ${\text{MoO}}_{3}\cdot$ H_2_O configurations are explored with models (e)–(j). Crystallographic parameters and relative energies according to *ab initio* modeling are collected in Table I.

**TABLE I.** Crystallographic parameters and relative energies (in kJ/mol per formula unit) for models (a)–(j) presented in Fig. 1. The relative energies are calculated independently for the β- ${\text{MoO}}_{3}\cdot 2$ H_2_O and β- ${\text{MoO}}_{3}\cdot$ H_2_O structures.

Hydration	β- ${\text{MoO}}_{3}\cdot 2$ H_2_O				β- ${\text{MoO}}_{3}\cdot$ H_2_O					
Model	(a)	(b)	(c)	(d)	(e)	(f)	(g)	(h)	(i)	(j)
Space group	$P2_{1}/n$	$P2_{1}/c$	*Pbca*	$P\bar{1}$	*P*1	$P2_{1}$	$P2_{1}/c$	$P2_{1}$	*P*1	$P2_{1}2_{1}2_{1}$
*a* (Å)	10.59	10.72	13.73	10.59	7.35	7.63	5.42	5.31	10.68	5.33
*b* (Å)	13.66	13.66	10.61	17.36	10.62	10.85	10.70	10.52	10.48	10.42
*c* (Å)	10.72	14.82	5.35	17.36	7.66	7.28	5.22	5.33	10.69	5.36
α (°)	90.00	90.00	90.00	103.67	90.00	90.00	90.00	90.00	90.00	90.00
β (°)	91.88	134.41	90.00	88.81	90.14	87.54	89.41	88.50	87.13	90.00
γ (°)	90.00	90.00	90.00	88.81	90.18	90.00	90.00	90.00	90.00	90.00
*Z*	16	16	8	32	8	8	4	4	16	4
ΔE (kJ/mol)	0.12	0.00	2.36	0.00	0.00	10.68	9.85	0.98	2.22	1.44
ZPE-corr (kJ/mol)	0.12	0.01	1.72	0.00	0.00	10.81	7.77	1.20	2.52	1.73

The basis for all considerations for the dihydrate structure is model (a), which corresponds directly to the structure proposed by Crouch and Baker,^31^ based on high-resolution NPD measurements of a perdeuterated specimen. This structure exhibits monoclinic $P2_{1}/n$ symmetry (*Z* = 16), which can be standardized to the $P2_{1}/c$ (*Z* = 16) projection (model b) without requiring any further symmetrization, aside from certain crystallographic coordinate permutations and changes in the orientation of lattice vectors. Moreover, the β-MoO_3_ lattice is only slightly distorted from the *Pbcm* symmetry (*Z* = 4), with the largest atomic displacements from the idealized structure within 0.75 Å. Upon inclusion of water molecules in the lattice, an aristotypic orthorhombic variant with the maximal *Pbca* (*Z* = 8) symmetry was identified (model c). Analysis of phonon band structure (see Fig. S3) reveals the presence of minor mechanical instabilities in each case. Frozen-phonon tracing using models commensurate with the wave vector indicates alternative supercell configurations. Among them, a fully mechanically stable, centrosymmetric supercell with *P* $\bar{1}$ (*Z* = 32) symmetry was identified.

**FIG. 2.**
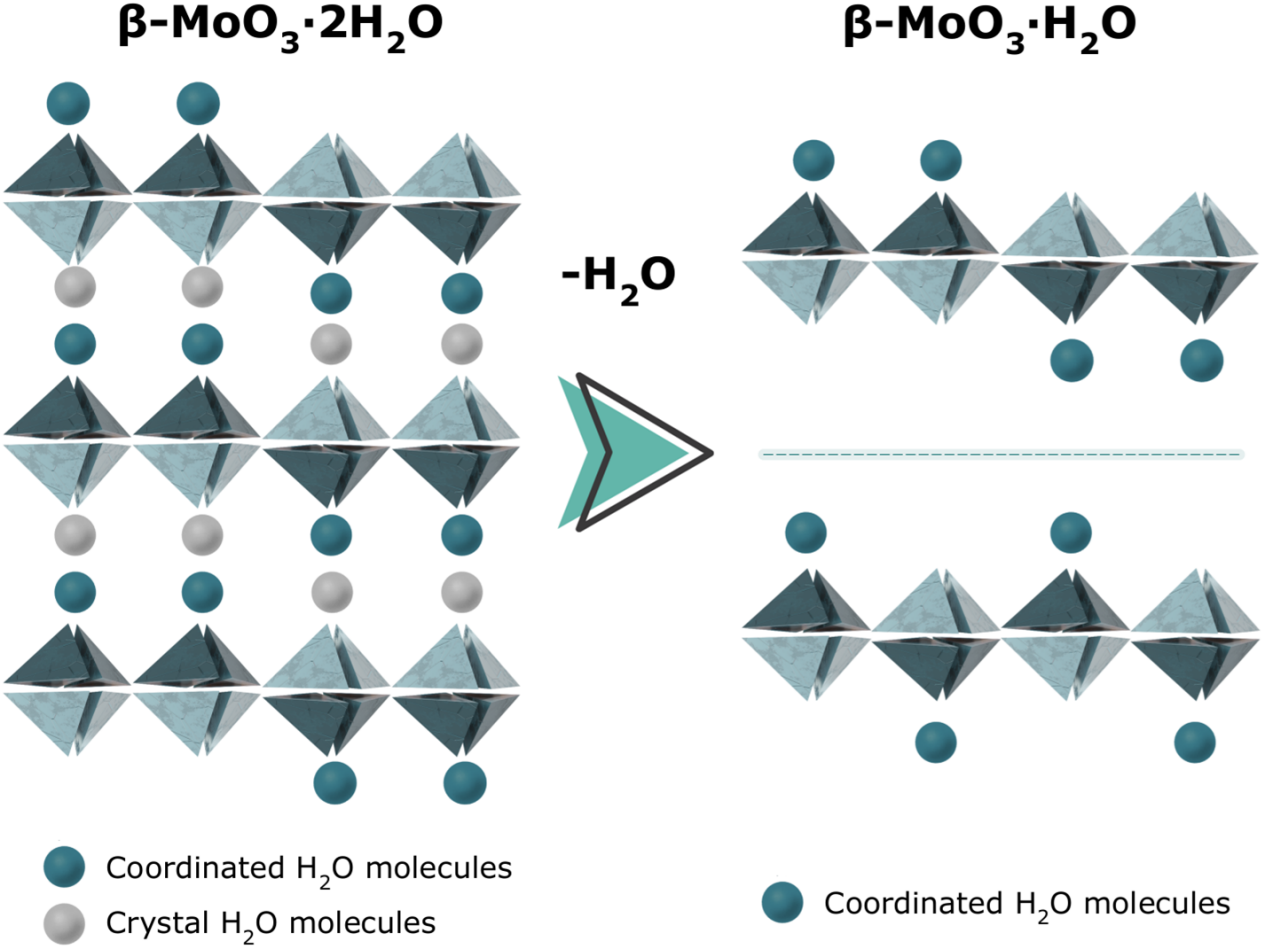
Schematic representation of the crystal structures of β- ${\text{MoO}}_{3}\cdot$ 2H_2_O (dihydrate) and β- ${\text{MoO}}_{3}\cdot$ H_2_O (monohydrate). The layered frameworks are built from MoO_5_ square pyramids, which coordinate additional water molecules (green spheres) on the opposite sites to form distorted MoO_6_ octahedra. Light-gray spheres represent hydrogen-bonded intercalated H_2_O molecules. Upon dehydration, β-MoO_3_ an elusive structure is formed, and two plausible configurations are schematically presented on the right. Adapted from Ref. 24.

The starting point for this analysis of the β– ${\text{MoO}}_{3}\cdot$ H_2_O framework is the recently reported *Virgilluethite* topology, in which the positions of water molecules were not determined.^24^ In order to account for this uncertainty, water molecules were placed according to a schematic hydration model from Fig. 2 (see bottom right panel). Subsequently, short, 5 ps NPT-BOMD simulations with Parrinello–Rahman barostat were conducted at the conventional cell level, resulting in a dynamically stable configuration. This structure was then accurately optimized at 0 K and subjected to phonon analysis, resulting in model (e) with *P*1 (*Z* = 8). Vibrational analysis across the FBZ reveals full mechanical stability as illustrated in Fig. S4. In order to screen potential alternative structural configurations for elusive  beta- ${\text{MoO}}_{3}\cdot$ H_2_O structure, model (f) was introduced, as characterized by a different arrangement of coordinated water molecules. This variation gave rise to a non-centrosymmetric, monoclinic structure with *P*2_1_ (*Z* = 8) symmetry.

Rietveld refinement attempts on a structurally analogous tungstite model by Szymanski ( $P2_{1}/c$; *Z* = 4)^75^ revealed a substantial positional disorder and a lack of long-range proton ordering. This geometry-relaxed configuration is designated as model (g), yet with pronounced mechanical instabilities manifested in the phonon band structure (see Fig. S4). Following the unstable phonon modes led to the identification of additional non-centrosymmetric structures, denoted as monoclinic model (h) with $P2_{1}$ (*Z* = 4) and its supercell (*Z* = 16) triclinic variant (i). Further symmetrization of model (h) results in the centrosymmetric $P2_{1}$ (*Z* = 4) structure denoted as model (j).

The conclusions drawn from the energy ranking presented in Table I indicate that the effect of *k*-point sampling, which cannot be entirely eliminated, remains within approximately 0.1 kJ mol^−1^, as demonstrated by the comparison between models (a) and (b), differing solely in their crystallographic conventions. The lattice distortion contributes an additional energy difference of approximately 0.5 kcal mol^−1^ in model (c), which is further stabilized by zero-point energy (ZPE). Furthermore, model (d) is found to be isoenergetic with model (b), although it is significantly more computationally demanding. Nevertheless, it produces an identical spectral profile to model (a), with minimal ZPE influence—comparable to that observed for models (a) and (b). In conclusion, the structure originally defined by Crouch and Baker is confirmed to be both mechanically reliable and energetically favorable and has been considered as the representative model of β-MoO_3_ · 2H_2_O for further scrutiny.^31^

In the case of MoO_3_ · H_2_O, the global minimum is defined by model (e), derived from the *Virgilluethite* topology and the AIMD simulations. Models (f) and (g) were found to be energetically unstable, whereas structures obtained from the frozen-mode analysis (h–j) yield configurations energetically proximate to the global minimum.

**FIG. 3.**
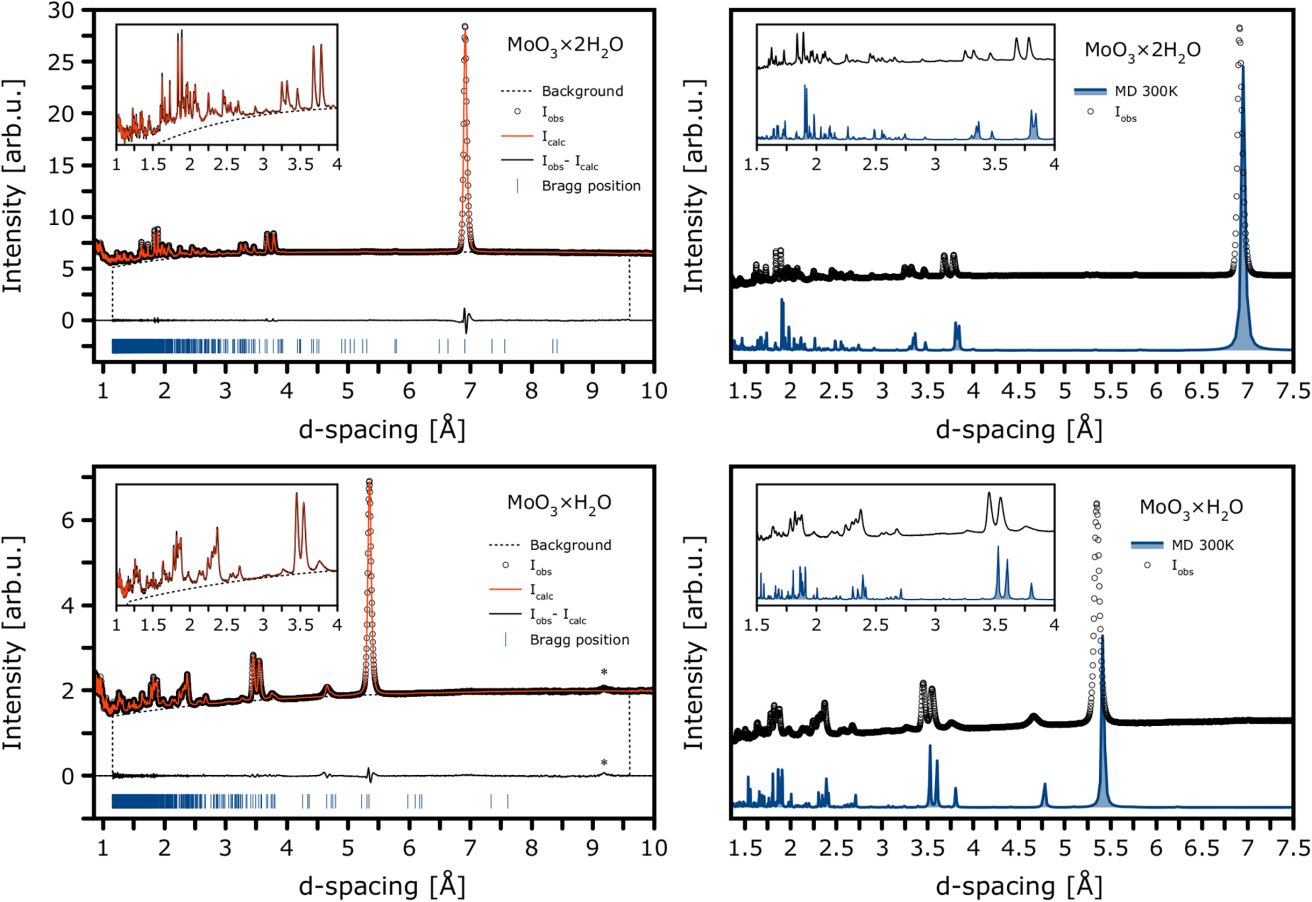
(Left) Le Bail fits to high-resolution NPD data recorded on cold-neutron WISH diffractometer (bank 3) over a wide *d*-spacing range for the most plausible structural models of ${\text{MoO}}_{3}\cdot 2$ H_2_O (top) and ${\text{MoO}}_{3}\cdot$ H_2_O (bottom), selected based on total-energy ranking and agreement factors (see the main text for more details). The asterisk in the bottom panel marks an unindexed reflection, suggestive of a possible superstructure formed in the monohydrate form. (Right) Simulated NPD patterns for both forms, generated from cumulative AIMD snapshots (NVT production runs following NPT equilibration at 300 K). The insets to all panels provide magnified views of the low-*d* spacing regime.

To select the most plausible models in comparison with the experiment, Le Bail analysis of the WISH data was employed, as summarized in Fig. 3 and Table II. Among the most representative and relevant candidate models, namely, (a), (b), (e), and (f) (the latter two being mechanically stable structures for the monohydrate), the dihydrate model (a), corresponding to the perdeuterated NPD structure established by Crouch and Baker,^31^ provides the best agreement for β- ${\text{MoO}}_{3}\cdot 2$ H_2_O, while the monohydrate model (e) emerges as the most consistent representation of β- ${\text{MoO}}_{3}\cdot$ H_2_O. This is corroborated by a more detailed, statistical insight reported in the supplementary material (Fig. S5 along with Tables S1 and S2, where the corresponding JANA2020 output files have also been deposited on Zenodo).

**TABLE II.** Crystallographic parameters obtained from Le Bail refinements of the high-resolution WISH neutron diffraction data for representative structural models of β- ${\text{MoO}}_{3}\cdot$ 2H_2_O and β- ${\text{MoO}}_{3}\cdot$ H_2_O. For reference, the mineral analogues *Sidwillite* ( β- ${\text{MoO}}_{3}\cdot$ 2H_2_O)^32^ and *Virgiluethite* ( β- ${\text{MoO}}_{3}\cdot$ H_2_O)^24^ are included from the literature. All data rounded to two decimals.

Model	(a)	(b)	Sidwillite	(e)	(h)	Virgilluethite
Space group	$P2_{1}/n$	$P2_{1}/c$	$P2_{1}/n$	*P*1	$P2_{1}$	$P2_{1}/c$
*a* (Å)	10.48	10.48	10.62	7.34	5.14	7.28
*b* (Å)	13.82	13.82	13.83	10.62	10.49	10.69
*c* (Å)	10.62	14.71	10.48	7.61	5.34	7.49
α (°)	90.00	90.00	90.00	89.70	90.00	90.00
β (°)	91.59	133.82	91.61	88.70	88.24	91.08
γ (°)	90.00	90.00	90.00	91.23	90.00	90.00
$R_{p}$	0.29	0.29	⋯	0.56	0.98	⋯
$wR_{p}$	0.47	0.48	⋯	0.63	1.53	⋯
GOF	2.27	2.29	⋯	1.85	5.27	⋯

The left-hand panels in Fig. 3 summarize the Le Bail refinements to the high-resolution WISH data that underpinned our final selection of the most credible structural models. In the following, we, therefore, focus on models (a) and (e), yielding superior agreement factors. The diffraction patterns recorded for β- ${\text{MoO}}_{3}\;\cdot$ H_2_O reveal pronounced peak broadening, consistent with a degree of disorder and possibly reduced symmetry, in full accordance with our rationale developed above. These results also support earlier spectroscopic fingerprints with IR and Raman, revealing broadening observed upon dehydration as reported by Séguin and Figlarz.^22^ Furthermore, we note an unindexed reflection at 9.2 Å, which may signal the presence of a superstructure.

The right-hand panels of Fig. 3 display the NPD patterns simulated for both models using atomic coordinates and unit-cell parameters extracted from cumulative geometries along the AIMD-NVT trajectories at 300 K. The monohydrate model reproduces the experimental diffraction exceptionally well, strongly supporting the proposed structural model of β-MoO_3_ · H_2_O. The provided topology supports outputs from an earlier neutron thermodiffractometry study performed on D1B (ILL) by Boudjada *et al.*^40^

**FIG. 4.**
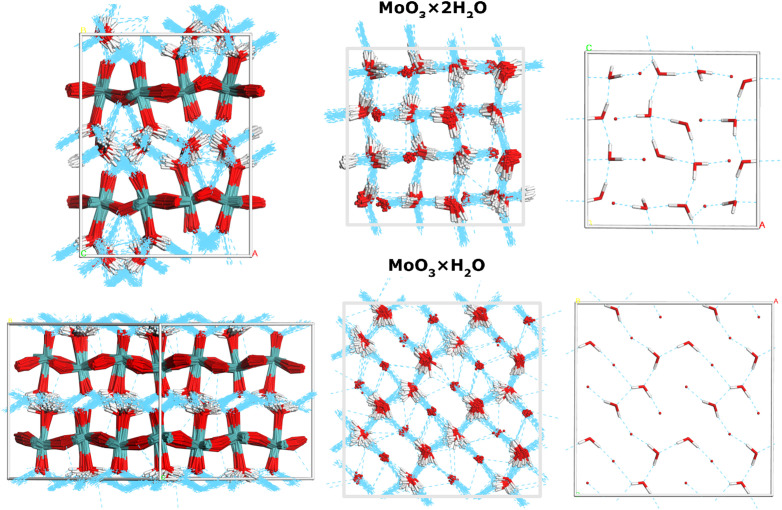
Cumulative configurations sampled from the NVT production runs for both representative models of ${\text{MoO}}_{3}\cdot 2$ H_2_O and ${\text{MoO}}_{3}\cdot$ H_2_O. The structures are shown in projections parallel (left) and perpendicular (middle) to the β-MoO_3_ layers, with the hydrogen-bond network highlighted in blue. (Right) Planar projection of the hydrogen-bond network derived from the static models relaxed at 0 K, with the β-MoO_3_ framework omitted for clarity.

Figure 4 presents the cumulative configurations for both systems, extracted from the MD snapshots along the NVT AIMD trajectories at 300 K. These are viewed parallel to the layers (left) and in top-down projection (middle), highlighting the time-evolved hydrogen-bond networks. These are compared to the static picture, derived from geometry optimization at 0 K (right). A closer look at the short-range intermolecular arrangement is also presented in Figs. S6 and S7 as compared to all models considered in the present work. In addition, an inspection of the pair-distribution functions at 50 and 300 K is shown in Fig. S8, providing a detailed insight into the close-contacts.

Altogether, the AIMD simulations indicate that the interlamellar water in β- ${\text{MoO}}_{3}\cdot 2$ H_2_O maintains a relatively ordered arrangement, characterized by a fully saturated hydrogen-bond donor–acceptor network. In contrast, the monohydrate exhibits a pronounced in-plane orientational disorder of water molecules. The results are reminiscent of those recently observed in tungstite.^60^ This contrast is corroborated by the spectroscopic fingerprints historically reported for the dehydration sequences in ${\text{MoO}}_{3}\cdot x$ H_2_O, where broadening of librational and O–H stretching bands accompanies the loss of order in the hydrogen sublattice.^22^ Importantly, the so-called “ice rules” are difficult to satisfy in both ${\text{MoO}}_{3}\cdot$ H_2_O and ${\text{WO}}_{3}\cdot$ H_2_O.^76^ We propose that breaking of these rules stays behind the observed diffraction peak broadening, and further highlights the role of the paddle-wheel mechanism in facilitating proton conductivity in ${\text{MoO}}_{3}\cdot$ H_2_O.^77^

### B. Phonon properties

**FIG. 5.**
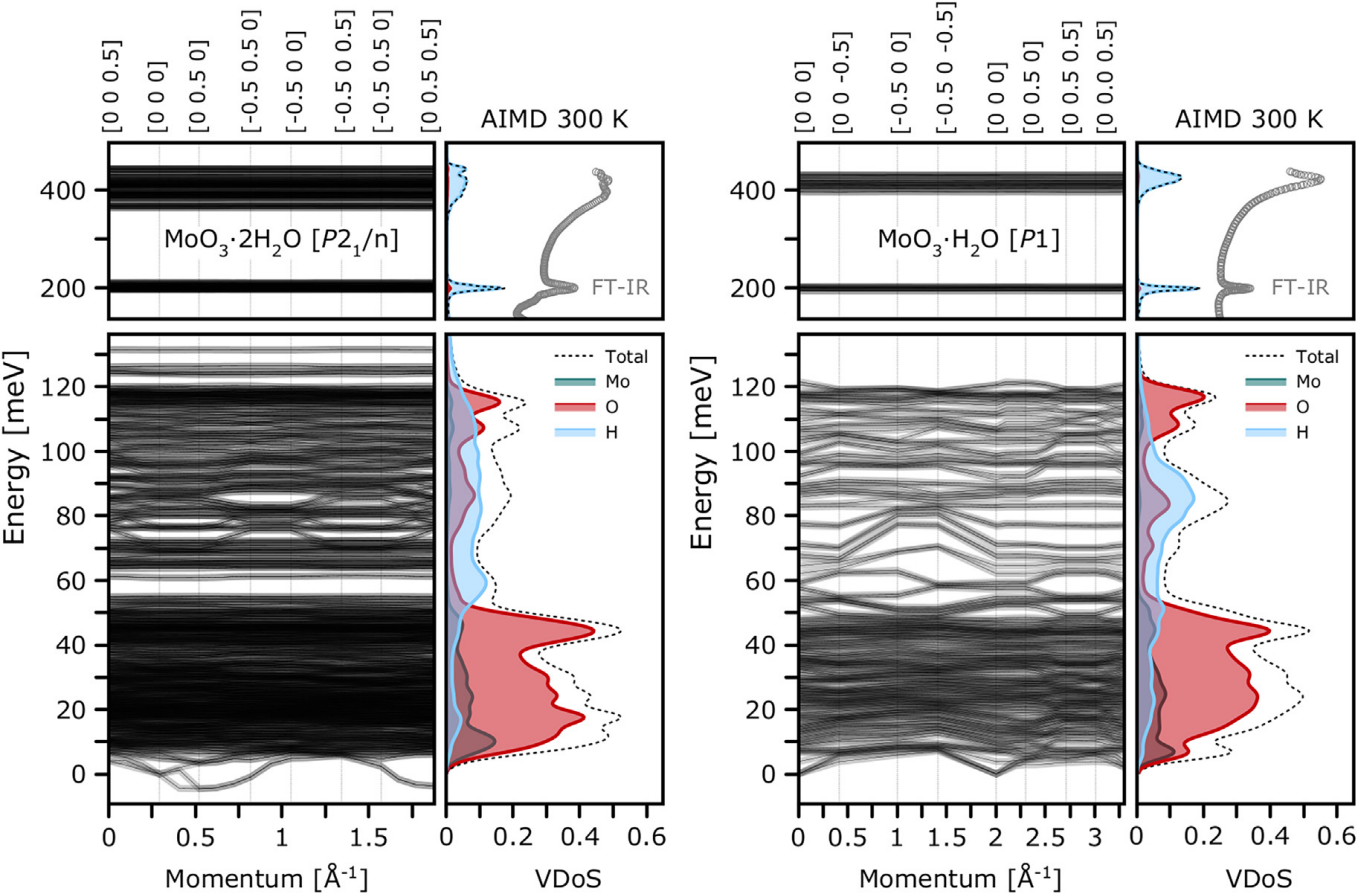
Phonon dispersion relations (left panels), obtained from harmonic lattice dynamics at 0 K (HLD) as compared to the vibrational densities of states (VDOSs) calculated at 300 K from the Fourier transform of the velocity–autocorrelation function (VACF) for the most credible structural models of β- ${\text{MoO}}_{3}\cdot 2$ H_2_O (left) and β- ${\text{MoO}}_{3}\cdot$ H_2_O (right). For comparison, the high-frequency IR response is presented for both materials, according to Günter.^20^

Figure 5 shows phonon band structure calculated with linear-response at 0 K alongside atom-projected VDOSs at 300 K for the most representative models of β- ${\text{MoO}}_{3}\cdot 2$ H_2_O (left) and β- ${\text{MoO}}_{3}\cdot$ H_2_O (right). In the dihydrate form, we observe a set of small (below 2 meV), imaginary modes beyond the Γ-point, in the vicinity of the [0 0.5 0] and [0 0.5 0.5] special points. As shown in Fig. S3(d), calculations within the symmetry-reduced 1 × 2 × 2 supercell with Z=32 ( $P\bar{1}$) yield a fully mechanically stable framework at 0 K, exhibiting an identical phonon band structure. This comparison highlights the crucial role of finite-temperature effects in stabilizing the closely related higher-symmetry $P2_{1}/n$ framework, which cannot be captured within the harmonic approximation at 0 K. The phonon dispersion relations calculated for the β- ${\text{MoO}}_{3}\cdot$ H_2_ framework show complete mechanical stability.

An inspection of the apVDOSs obtained from AIMD simulations at 300 K reveals only a weak temperature-dependence of the underlying band positions. Comparison with the room-temperature IR spectra reported by Günter^20^ underscores both the robustness of the adopted methodology and the high reliability of the structural models considered in this work. In particular, the simulations reproduce with high fidelity the δ(OH) bending mode (ca. 200 meV) and the ν(OH) stretching band (ca. 400 meV), the latter being captured with an excellent description of its characteristic band shape.

**FIG. 6.**
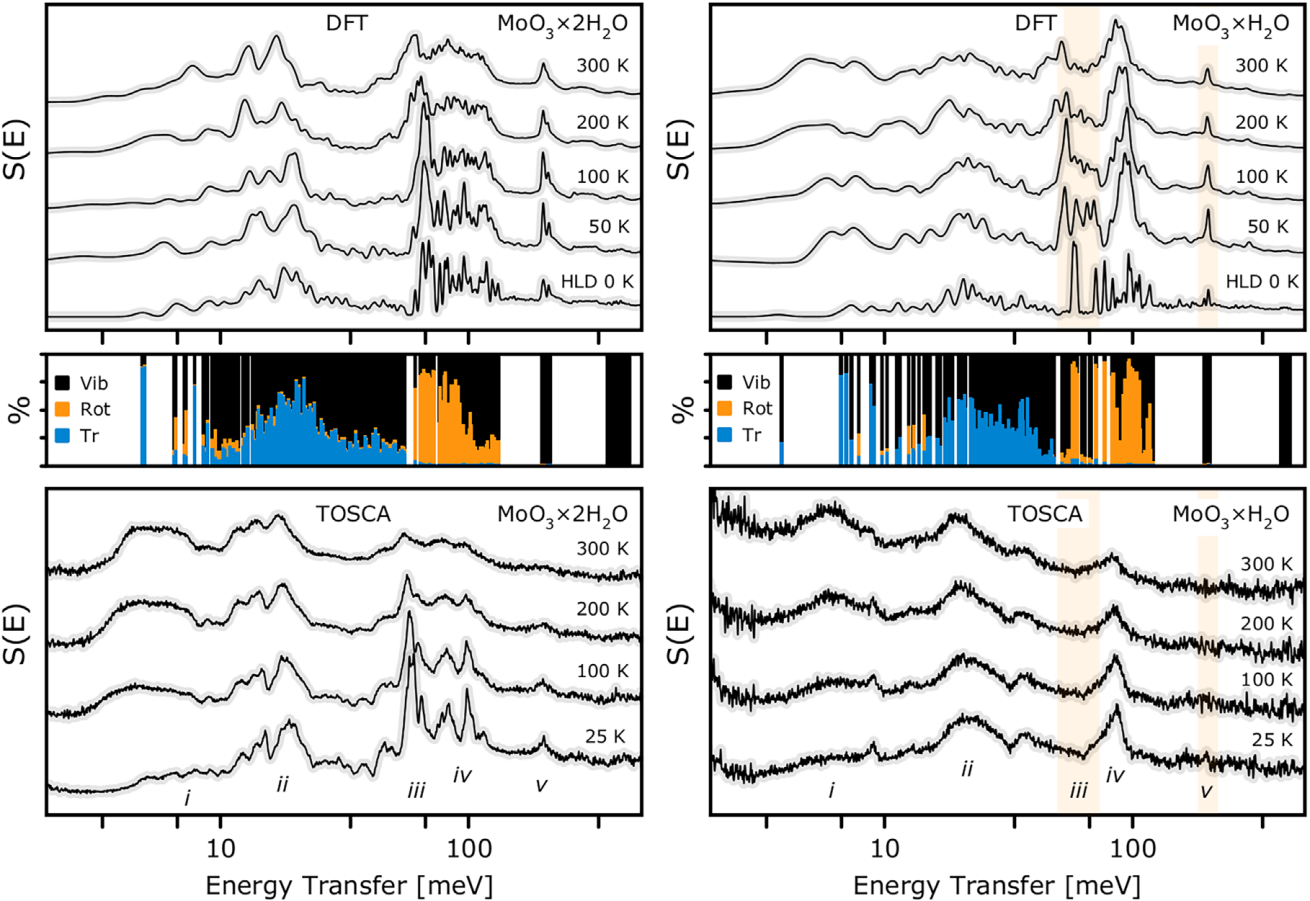
Temperature-variable INS spectra for β- ${\text{MoO}}_{3}\cdot 2$ H_2_O (left) and β- ${\text{MoO}}_{3}\cdot$ H_2_O (right), recorded on the TOSCA spectrometer between 25 and 300 K (bottom panels), alongside 0 K theoretical spectra (top panels) and a decomposition of the normal modes into translational, librational (rotational), and intramolecular contributions of the water molecular centroids (middle panels). The 0 K spectra and mode decomposition are obtained within harmonic lattice dynamics (HLD), whereas the finite-temperature spectra are simulated from AIMD trajectories. Shaded bands mark spectral regions that appear smeared in the experiment relative to the harmonic model, consistent with orientational disorder of water molecules.

Further inspection of the VACF-projected VDOSs allows for a detailed insight into the nuclear-specific contributions. The Mo-related vibrations are confined below 50 meV. The Mo–O vibrations are distributed similarly in both hydrates, as reflected in very similar oxygen-projected VDOSs. A precise assignment of the β-MoO_3_ framework has been presented in the literature (see, e.g., Refs. 22 and 78). The largest differences originate from proton dynamics. The broader band shape near 400 meV naturally reflects the presence of two ensembles of hydrogen-bonded water molecules. In particular, a pronounced difference is seen in the 50–150 meV region. To scrutinize the origin of these differences further, we refer to the INS experiments, performed on an indirect-geometry TOSCA spectrometer (see Fig. 6).

The bottom panels in Fig. 6 show the temperature-dependent INS spectra, revealing that the band positions hardly vary with temperature, consistent with the low thermal cell expansion reported in Table S3. The temperature changes are mainly associated with an increasing population of the low-energy external modes, while the overall band structure is preserved up to ambient conditions. This indicates the absence of structural transitions across the temperature range relevant to the present work. It is important to note that, in contrast to the dihydrate, no subtle band structure is observed in the INS spectra below 100 K. This indicates that the orientational disorder of water is not merely a dynamic effect but persists even under cryogenic conditions.

Earlier INS studies of molybdenum oxides have been restricted to the hexagonal phase,^19^ whose spectra differ markedly from those observed here. By contrast, the monohydrate spectrum brings similarity to the recently reported data on tungstite ( ${\text{WO}}_{3}\cdot$ H_2_O).^60,79^

The results of both static and time-evolved simulations of the INS spectra are presented in the upper panels. These include the zero-temperature harmonic spectra together with the temperature-dependent INS spectra from AIMD simulations, accounting for the characteristics of the TOSCA spectrometer (see the top panel in Fig. 6). Notably, an excellent agreement is observed between the experimental and theoretical spectra for the dihydrate sample. The middle panels of Fig. 6 show the decomposition of the Γ-point modes for both systems of interest into translational, librational, and intramolecular contributions. Based on this decomposition, the spectra can be broadly divided into five characteristic regimes (i)–(v), each of which appears visibly broadened in the case of β- ${\text{MoO}}_{3}\cdot$ H_2_O. Apart from the broadening, in both cases, the lower part of the spectra (below ca. 33 meV) is dominated by host vibrations, namely, the shearing and deformation modes of β-MoO_3_ of a similar nature, where the water translations contribute through hydrogen projections as riding modes (i)–(ii). In line with the findings from the high-energy IR response, which is hardly accessible in the present INS experiment, this points to a comparable strength of the hydrogen bonding, since the H_2_O translations reflect the hydrogen-bridge ν(O … O) stretching modes. The primary spectral difference is observed in the range of ca. 40–140 meV (iii)–(iv), which can be clearly assigned to water librations. In particular, the characteristic in-plane librations, τ(H_2_O) (iii), of the coordinated water molecules are predicted to appear as a well-defined band at 60 meV. However, this feature is not detected experimentally, indicating the absence of a single, well-defined in-plane orientation of the water molecules. The upper component (iv), with a maximum around 50 meV, corresponds to the out-of-plane γ(H_2_O) deformation modes. Similarly, for β- ${\text{MoO}}_{3}\cdot$ H_2_O, no well-defined band is observed at 200 meV, corresponding to the δ(H_2_O) mode, which is likewise highly sensitive to the in-plane hydrogen-bond geometry.

### C. Neutron Compton scattering

Before we delve into the analysis of the results of the NCS experiments on β-MoO_3_ · H_2_O and β-MoO_3_ · 2H_2_O at 50, 100, 200, and 300 K, it is worth summarizing how the NCS observables inform us on the physical and chemical properties of condensed-matter systems. In doing so, we will concentrate on the proton and use a qualitative rather than quantitative description in order to build intuition to help us understand the properties of the hydrogen sublattice in hydrated molybdenum trioxides.

Usually, two sets of NCS observables, the widths (standard deviations) of nuclear momentum distributions (NMD widths) and the values of the nuclear kinetic energies, *Ekin*, are used as sources of information about the nuclear chemical dynamics in solid-state systems and molecules.^48–50^ Yet, these two types of observables do not seem to receive the same amount of attention in the literature, and the reason for this is the rather less intuitive nature of the first above-mentioned observable.^80^

First of all, the NMD widths are quantum observables pertaining to momentum space, where concepts, such as atomic displacement, PES, and turning points can, in principle, be formulated mathematically, but in a much less intuitive manner than their counterparts in position space.^80^ What comes to the rescue here, especially in the case of the proton, is the Heisenberg uncertainty principle. For protons, the apVDOSes have centers of gravity (CoGs) at vibrational energies much higher than the thermal energy at room temperature, and consequently, the proton wave functions are, even in crystals at room temperature, in the ground state for a given PES.^48–50,80^ Consequently, the widths of the proton distributions, given by the squares of the moduli of the proton wave functions in momentum space, are inversely proportional to the positional uncertainties of protons around the loci of the minima of local PESs, measured by atomic displacement parameters (ADPs). This inverse proportionality holds for protons in condensed-matter systems up to room temperature and beyond.^80^

Thus, unless the local PES changes shape, the value of the proton NMD width for this underlying PES is not expected to change. Following the uncertainty principle, one can say, that the more a proton in a given PES is delocalized in space, the less it is “localised in momentum space” (i.e., the narrower its NMD width), and vice versa. Importantly, a question remains: what causes the shape change of the local PES? It can be caused by a phase or glass transition or by a modification of the apVDOS shape with temperature due to the hardening or softening of selected vibrational modes, resulting in a net effect of shifting the CoG of the apVDOS.^48–50,80,81^

What other quantum physical phenomena inform the value of the NMD width and what other quantum observables are related to it? An important property of a quantum particle in a local PES is the restoring force acting on it toward the equilibrium position. It describes the force with which an atom is held together in a crystal or a molecule, informing us about the stiffness of the local chemical bond or the degree of physical confinement the atom is subject to. It can be shown that, even for PESs possessing a relatively large amount of anharmonicity (with cubic and quartic terms on the order of 10% of the harmonic term,^82–84^) the magnitude of the effective restoring force constant, *k*, related to the NMD width is relatively close to its counterpart given by the harmonic approximation.^50,80,85,86^

There are two important consequences of this relatively good performance of the harmonic approximation. First, the value of *k* can be well approximated as being proportional to the square root of the effective frequency of vibration, ω, given by the CoG of the apVDOS at a given temperature. This effective vibrational frequency informs us about the local curvature of the effective PES, calculated in the spirit of the mean-field approximation for a nucleus of mass *M*, $\frac{\omega }{\hbar }=\sqrt{\frac{k}{M}}$. Second, by virtue of the virial theorem for a quantum harmonic oscillator, the nuclear kinetic energy, *Ekin*, for a given nucleus of mass *M* in its ground state, is equal to half of its zero-point energy, $\frac{\omega }{2}$, i.e., $\text{Ekin}=\frac{\omega }{4}$, a property that is very well fulfilled for hydrogen in condensed-matter systems.^48–50,80^ Taken together, in this mean-field picture, for a given atomic species, $k∼\sigma^{1/4}$, which provides a direct link between the width of the nuclear momentum distribution and the local physical/chemical environment of atomic species.^48–50,80^ Importantly, from the point of view of the NCS technique, it is almost impossible to tell whether the physical confinement or the local chemical bond strength dictates the magnitude of the NMD width, and only with the help of advanced *ab initio* modeling can one provide an answer.

Finally, the NCS observable of the nuclear kinetic energy, although more intuitive than the NMD width, still requires further elucidation in certain contexts. One of these is the correlation between the topology of the local physical confinement of a nucleus and the value of *Ekin*. It has been shown that in systems where protons are physically confined in space, such as in water confined in carbon nanotubes,^87–89^ water protons in protein hydration shells,^90,91^ structural protons in silica xerogels,^92^ water confined in silica nanopores,^93^ water in the pores of the beryl mineral,^94^ and 2D-nanoconfined water and ice adsorbed in graphene oxide sponges,^95^ the values of *Ekin* of protons are lower than their counterparts representing the local nuclear dynamics of protons in a three-dimensional network of hydrogen bonds in bulk water. Assuming that the value of *Ekin* of a proton is proportional to the effective dimension of the hydrogen-bond network the confined water is involved in, one can attempt to assign an effective (fractal or Hausdorff) dimension of this network to a given value of the kinetic energy. For example, in the case of water protons in carbon nanotubes, the experimental value of *Ekin* is ca. 100 meV,^87–89,94^ compared to its counterpart in bulk water of 143 meV,^96^ thus giving the effective dimension of 2.12, which confirms the two-dimensional nature of the water bond network in these systems. Interestingly, the confinement-induced lowering of the value of *Ekin* of water protons can be explained by a concomitant downshifting of the CoG of the proton apVDOS in water caused by softening of vibrational modes.^81^

Equipped with the basic rules of thumb pertaining to the local proton chemical dynamics as observed by the NCS, we can proceed to the discussion of the proton NMD kinetic energy values obtained in hydrated molybdenum trioxides.

**FIG. 7.**
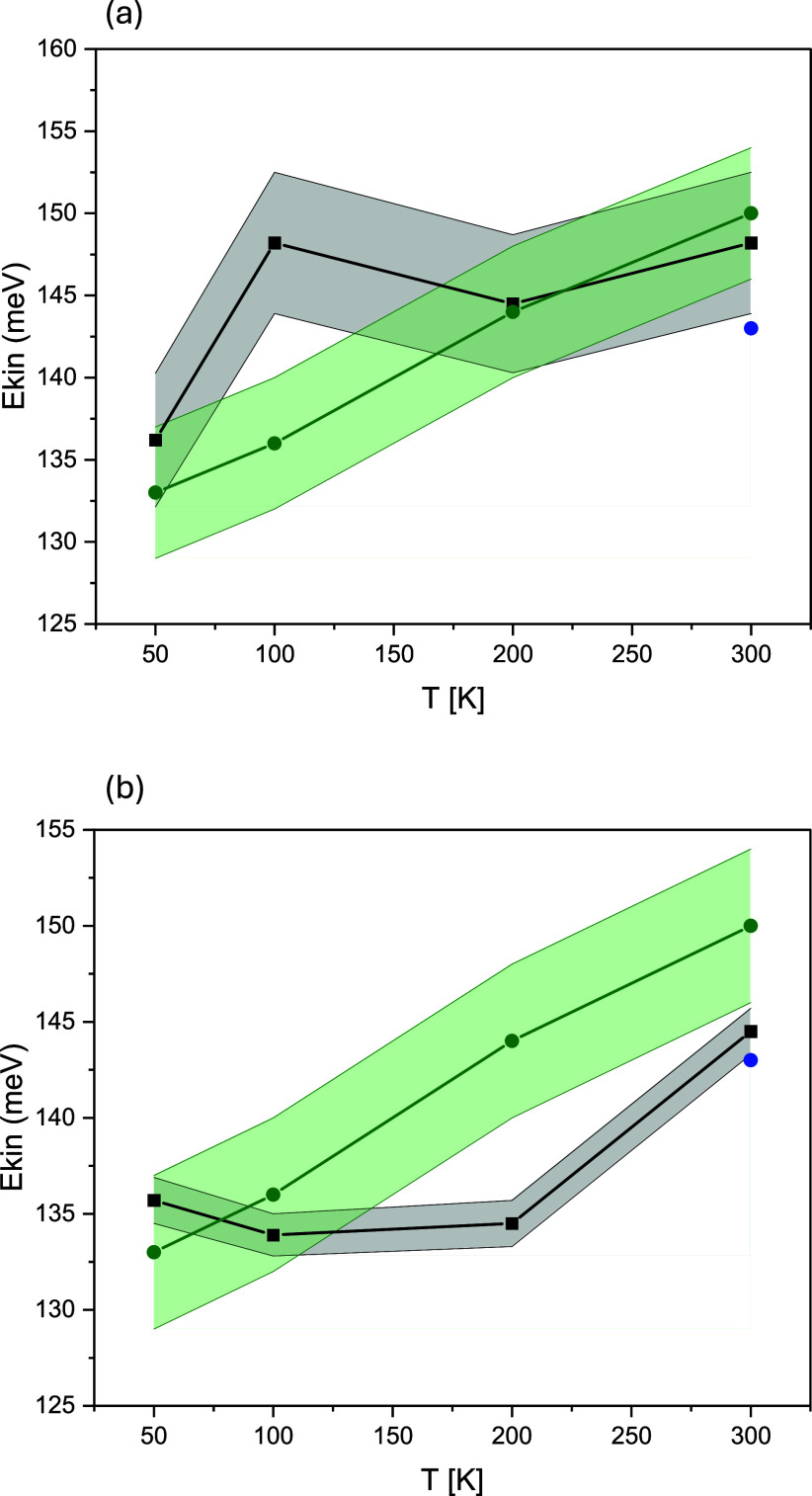
The fitted values of the proton kinetic energy for β-MoO_3_ · H_2_O [panel (a)] and β-MoO_3_ · 2H_2_O [panel (b)] samples at 50, 100, 200, and 300 K are shown. The gray and green error-band plots represent the experimental values and the values obtained by Maiti *et al.*^81^ using classical MD simulations with the TIP4P-2005f model for water inside a single-wall carbon nanotube of diameter 8.1 Å [(6,6) chirality SWCNT]. The solid blue points show the value of the proton kinetic energy in bulk water at 300 K as reported by Pietropaolo *et al.*^96^

The first observation that comes to mind immediately after the inspection of Fig. 7, showing the fitted values of the proton kinetic energy for β-MoO_3_ · H_2_O [panel (a)] and β-MoO_3_ · 2H_2_O [panel (b)] samples at 50, 100, 200, and 300 K, is that, at 300 K, the *Ekin* of protons in both systems reaches a value equal, within one standard deviation (1-STD), to that of the proton kinetic energy in bulk water at 300 K as reported by Pietropaolo *et al.*^96^ Using the dimensionality argument, one can conclude that, in the limit of room temperature, protons in both systems behave like protons within a three-dimensional network of hydrogen-bonded molecules in bulk water. Moreover, this argument is consistent with the picture in which, at 300 K, the three-dimensional network of bonds in which protons in both systems are embedded yields an effective PES with a similar curvature to that of bulk water, with similar effective (mean-field) force constant magnitudes.

Interestingly, the inspection of Fig. 4 reveals similarities between the AIMD-simulated apVDOSes of protons in both hydrated molybdenum trioxides and their counterparts obtained from the AIMD simulation of bulk water at room temperature^97^ and classical MD simulation of water confined in a single-wall carbon nanotube (SWCN) of a diameter of 13.6 Å and a (10,10) chirality, and a diameter of 8.1 Å and (6,6) chirality, as well as in Ih ice.^81^ All molecular dynamics simulations can fully resolve the band centered at around 1700 cm^−1^, attributable to the water bending mode, which is well separated from the band between 3200 and 3800 cm^−1^ with not-fully resolved components due to asymmetric and symmetric O–H stretching. Below ca. 1000 cm^−1^, two separate bands due to external modes of translation and libration are visible in simulations of protons in bulk water^97^ and in Ih ice.^81^ In the case of the apVDOS of protons in water confined in the SWCN of a diameter of 13.6 Å and a (10,10) chirality, these two bands merge, similar to the case of both hydrated molybdenum trioxides, while in the case of the protons in water in the SWCN of a diameter of 8.1 Å and (6,6) chirality, three distinct bands centered at 200, 500, and 800 cm^−1^ are clearly visible.^81^

Taken together, the similarities in the shapes of proton apVDOSes in hydrated molybdenum trioxides and bulk water at 300 K lead to similar CoGs of their vibrational densities of states and, consequently, the values of the proton kinetic energies at 300 K. The slightly higher value of *Ekin* of protons in β-MoO_3_ · H_2_O at 300 K, compared to β-MoO_3_ · 2H_2_O, most likely stems from a slightly higher intensity of the proton apVDOS in β-MoO_3_ · 2H_2_O between 480 and 800 cm^−1^, which shifts the CoG of the proton apVDOS toward lower energies.

At 50, 100, and 200 K, the proton apVDOSes in β-MoO_3_ · H_2_O obtained from the AIMD simulations performed for the structure described by model (e) (data not shown) exhibit three distinct bands centered at ca. 160, 400, and 720 cm^−1^, which clearly resemble their counterparts visible in the molecular dynamics simulation of the protons in water in the SWCN of a diameter of 8.1 Å and (6,6) chirality. Additionally, all bands, due to the bending and stretching modes present in both systems, are very similar. Consequently, the CoGs of the proton apVDOSes are similar in both systems, and they integrate to very similar values of *Ekin*. The only discrepancy between the values of *Ekin* of protons in β-MoO_3_ · H_2_O and protons in water in the SWCN of a diameter of 8.1 Å and (6,6) chirality, visible at 100 K, is most likely due to subtle differences in the fine structure of the intra- and intermolecular vibrational bands in the apVDOSes.

In the case of protons in β-MoO_3_ · 2H_2_O, the AIMD simulation performed for the structure described by model (a) at 200 K yields a proton apVDOS (data not shown), in which, below 1000 cm^−1^, only two bands can be clearly separated: a narrow band at ca. 150 cm^−1^ and a wideband at ca. 420 cm^−1^. Additionally, a band at ca. 1600 cm^−1^ due to the intramolecular bending mode is present, and the stretching band, unlike in β-MoO_3_ · H_2_O, is split into two sub-bands, one broad band centered at ca. 3200 cm^−1^, and another narrow band centered at ca. 3500 cm^−1^. Taking into account that high-frequency vibrational modes carry the highest amount of kinetic energy, and thus contribute most to the total value of *Ekin*, this explains why the values of *Ekin* of protons in β-MoO_3_ · 2H_2_O and protons in water in the SWCN of a diameter of 8.1 Å and (6,6) chirality differ quite markedly at 200 K.

At 50 and 100 K, the three-band picture below 1000 cm^−1^ is restored in β-MoO_3_ · 2H_2_O, with one narrow band at 150 cm^−1^, followed by another narrower band at 520 cm^−1^, and a wideband centered at ca. 720 cm^−1^ (data not shown). Additionally, the bending mode band at ca. 1600 cm^−1^ is now split into two sub-bands separated by ca. 80 cm^−1^ in energy, and the stretching band is now split and exhibits multiple peaks grouped into two bands, one centered around 3200 cm^−1^ and another around 3500 cm^−1^. The net result of this structure of the proton apVDOSes is that the values of the *Ekin* of protons at 50 and 100 K are very similar to their counterparts in water protons confined in the SWCN of a diameter of 8.1 Å and (6,6) chirality.

Interestingly, below 300 K, there is a clear distinction between the temperature dependence of the values of the *Ekin* of protons in water confined in the SWCN of a diameter of 8.1 Å and (6,6) chirality and protons in water confined in a single-wall carbon nanotube (SWCN) of a diameter of 13.6 Å and a (10,10) chirality (see Fig. 4 in Ref. 81). While in the former case, there is a monotonic increase in the value of *Ekin* with temperature, in the latter case, the values of the *Ekin* are almost constant and equal to the value of the *Ekin* of protons in bulk water at 300 K. This marked difference in the behavior of protons in water confined in SWCNs, depending on their diameter and chirality, can be traced back to the differences in their respective proton apVDOSes (see Fig. 6 in Ref. 81). Namely, in the case of protons in water confined in an SWCN of a diameter of 13.6 Å and a (10,10) chirality, both intramolecular water bending and stretching bands are much broadened and shifted toward higher energies, thereby shifting the CoG of the proton apVDOS upward and increasing the value of *Ekin* with respect to the protons in water confined in SWCNs of smaller diameter. Moreover, because the Bose–Einstein statistics governing the occupancies of the phonon modes ensure that the modes whose population changes the least with temperature are those with the highest energy, any proton apVDOS in which the high-energy modes dominate will yield virtually temperature-independent values of proton kinetic energy.

Beyond the apVDOS picture, this marked difference in the temperature behavior of the value of proton *Ekin* depending on the nanotube diameter is a reflection of the fact that, at higher SWCN pore diameters, the vibrations of water molecules are more mechanically decoupled from the vibrations of the SWCN skeleton and more independent of the topology of confinement. In simple terms, the water confined in larger pore volumes resembles bulk water more than the water confined in small-pore-size geometries. It should, thus, be naturally expected that protons in water molecules confined in β-MoO_3_ · H_2_O should have more temperature-dependent values of their kinetic energy than their counterparts in β-MoO_3_ · 2H_2_O due to a greater degree of confinement (smaller interlamellar space of the β-MoO_3_ layers). Indeed, the temperature dependence of proton kinetic energy in β-MoO_3_ · 2H_2_O is more constant between 50 and 200 K than its counterpart in β-MoO_3_ · H_2_O. However, the local dynamics of two populations of water confined within the interlamellar space of the β-MoO_3_ layers in β-MoO_3_ · 2H_2_O are different from the dynamics of a single water layer confined in the SWCN of a similar diameter, and, as a result of these different dynamics, the temperature dependencies of the respective kinetic energy values of protons in these two systems are not identical.

As far as the connection between the values of *Ekin* of protons and the dimensionality of the hydrogen-bonded network of water molecules present in the hydrated molybdenum trioxides under investigation is concerned, it is perhaps more proper to speak about an effective dimension that corresponds to a motionally averaged physical dimension at a given temperature. Thus, when connecting the values of the proton kinetic energy to the local topology of hydrogen bonds, one has to think more in terms of the picture provided by our AIMD simulation in Fig. 4, rather than a zero-Kelvin-limit picture in Fig. 1. The topology of the hydrogen bonds in β-MoO_3_ · H_2_O and β-MoO_3_ · 2H_2_O provided by the AIMD simulation in Fig. 4 tells us that, in β-MoO_3_ · H_2_O, the ice rule of four hydrogen bonds per water molecule is not fulfilled at low temperatures, and with the increase in temperature, the water molecules in β-MoO_3_ · H_2_O tend to perform local motions in the attempt to fulfill this rule. As a result of this tendency, the motionally averaged density of local hydrogen bonds in β-MoO_3_ · H_2_O in the direction perpendicular to the β-MoO_3_ layers increases with temperature, and at room temperature, it resembles much more the fully developed three-dimensional hydrogen-bond network of water molecules in bulk water than is the case at 50 K.

The MCMC analysis of the proton recoil peaks in both hydrated molybdenum trioxides established that the bivariate Gaussian NMD describes the shapes of the proton recoil peaks in both systems at all temperatures more accurately than an isotropic Gaussian distribution. As evidenced by the trends clearly visible in Fig. 7 and our additional analysis of the bivariate Gaussian NMD provided in Figs. S10–S13 in the supplementary material, with the increase in temperature, the spatial distribution of the *Ekin*, measured in terms of the anisotropy of the kinetic energy tensor,^98^ and its isotropic average value in β-MoO_3_ · H_2_O and β-MoO_3_ · 2H_2_O converge to the values characteristic of proton kinetic energy in bulk water at room temperature in slightly different ways. In β-MoO_3_ · H_2_O, there is a sudden jump in the value of the isotropic average of the *Ekin* already at 100 K, and then the *Ekin* value remains almost constant and equal to its counterpart in bulk water at room temperature. In contrast, in β-MoO_3_ · 2H_2_O, the value of the isotropic average of the *Ekin* stays at the level of the *Ekin* of protons in water inside a single-wall carbon nanotube of a diameter of 8.1 Å [(6,6) chirality] from 50 up to 200 K, and then it jumps to the value characteristic of bulk water protons at room temperature.

Due to the fact that experimental data do not seem to contain enough information to be able to be fitted using tri-variate Gaussian momentum distributions, we have averaged the first two *ab initio*-simulated eigenvalues of the kinetic energy tensors, while keeping the third one, and compared, thus, obtained theoretical predictions for the longitudinal and perpendicular components of the proton NMDs to their counterparts obtained from the bi-variate Gaussian fits to the experimental data (denoted as red and blue solid data points with error bars in Figs. S12 and S13 in the supplementary material, respectively).

The fitted widths (standard deviations) of the bivariate longitudinal proton momentum distributions in β-MoO_3_ · H_2_O at 50, 100, 200, and 300 K are contrasted with the results of the *ab initio* simulations in Fig. S12 in the supplementary material. It is clearly visible from the inspection of this figure that the longitudinal component grows by a factor of two in an almost linear fashion with temperature, while the perpendicular component is practically temperature independent, with the NMD width at the level of three times the value of the longitudinal component at 50 K. In the case of β-MoO_3_ · 2H_2_O (see Fig. S13 in the supplementary material), the trend for the perpendicular component is the same as in β-MoO_3_ · H_2_O, but the longitudinal component stays almost constant between 50 and 200 K and then jumps at 300 K.

In light of what we said above about the temperature dependencies of the isotropically averaged values of *Ekin* of protons visible in Fig. 7, Figs. S12 and S13 in the supplementary material provide additional insight. The tendency of the protons in β-MoO_3_ · H_2_O to fulfill the ice rule can be clearly identified as the thermally activated motion in the direction perpendicular to the β-MoO_3_ layers, captured by the longitudinal component of the bivariate momentum distribution, while the perpendicular component captures the almost constant average effective bond strength experienced by the protons in water molecules confined within the interlamellar space of the β-MoO_3_ layers.

In the case of protons in β-MoO_3_ · 2H_2_O (Fig. S13 in the supplementary material), a similar assignment of the components of the bivariate momentum distribution is valid, and the longitudinal component captures the tendency of the water molecules to mimic the fully developed three-dimensional network of hydrogen bonds in bulk water at room temperature.

In both cases, the assignment of the two components of the bivariate momentum distribution is consistent with the temperature-dependent INS spectra. The thermally activated smearing out of the librational bands dictates the temperature dependence of the longitudinal component. The relatively thermally stable network of hydrogen bonds in water molecules within the interlamellar space appears to be responsible for the almost temperature-independent bands visible in the INS spectra above the librational bands, which underlie the perpendicular component of the bivariate momentum distributions in both systems.

Finally, we note that for both β-MoO_3_ · H_2_O and β-MoO_3_ · 2H_2_O, the AIMD simulations perform slightly better than the HLD simulations in mimicking the trends observed in the experimental components of the bivariate Gaussian momentum distributions. This is an expected result, given that AIMD naturally captures anharmonic effects that dictate changes in the shapes of the proton apVDOSes with temperature. Interestingly, in the particular case of the hydrated molybdenum trioxides under consideration, the net effect of mode softening/hardening with temperature on the positions of the CoGs of the proton apVDOSes in both systems is relatively small, and the major factor shaping the proton apVDOSes with temperature is the Bose–Einstein phonon population factor, which is applied as a multiplicative factor in the *ab initio* modeling protocol only after the apVDOSes have been calculated using HLD or AIMD. This explains the similarities between the temperature dependencies of the values of *Ekin* and the proton NMD widths as obtained from the HLD and AIMD simulations. In this context, it is important to note that both HLD- and AIMD-based simulation protocols yield apVDOSes by treating nuclei as classical objects, and only a fully quantum path-integral molecular dynamics simulation can treat both nuclei and electrons on equal footing and calculate the interatomic forces and the trajectories of nuclei by solving the electronic density problem and the nuclear motion quantum mechanically. It is, thus, recommended that, in the future, a path-integral–based simulation be employed for the calculation of both proton apVDOSes and proton kinetic energies and NMD widths in hydrated molybdenum trioxides.

## IV. CONCLUSION

This work has demonstrated the vast and largely untapped potential of global research protocols that combine neutron probes of structure and dynamics across different spatiotemporal scales, spanned by various scattering and diffraction techniques applied concurrently and augmented by *ab initio* modeling.

The *ab initio*-augmented structural search and refinement presented here serves as the first example of the powerful synergies between different experimental techniques and modeling. In the case of powder samples of condensed-matter systems containing protons in confined water, the application of powder neutron diffraction alone would have led to much more ambiguous results, were it not for the fact that *ab initio* modeling provided the initial clues for the preselection of candidate structures for further neutron diffraction–based structural elucidation.

The second example is provided by the synergies created between INS and NCS spectroscopies on the one hand and *ab initio* modeling on the other. The concurrent employment of the INS and NCS techniques, augmented by *ab initio* modeling, has highlighted the interplay between thermal effects and confinement-induced local proton dynamics in both hydrated molybdenum trioxides. The atom-projected vibrational densities of states of protons obtained from the HLD and AIMD simulations have proven to be powerful tools in the comparative analysis of the local nuclear dynamics of protons in hydrated molybdenum trioxides, in water confined in single-wall carbon nanotubes, and in bulk water. The dissection of the proton apVDOSes into contributions from translational, librational, and vibrational modes has provided valuable insights into the confinement-induced changes in the INS and NCS spectra and has allowed the identification of modes that respond directly to the geometry of confinement and those that are more resistant to constraints imposed by the local geometry and temperature effects.

The bivariate Gaussian modeling of the proton momentum distributions, employed successfully here for the first time in systems where water protons are confined, has provided a framework for a more precise assignment of translational, librational, and vibrational modes to specific geometric components of the proton momentum distributions in both systems and has shed light on the topology of the motionally averaged effective network of hydrogen bonds in the presence of different degrees of confinement.

The analysis has revealed that the longitudinal component of the nuclear kinetic energy of protons in water confined in MoO_3_ · H_2_O responds more strongly to the combined effects of temperature and confinement than its counterpart in MoO_3_ · 2H_2_O, which is most likely a manifestation of the natural tendency of the proton pool in the former system to fulfill the ice rules. This finding paves the way for future applied research in systems where the engineering of nuclear kinetic energy is of technological relevance.

## SUPPLEMENTARY MATERIAL

See the supplementary material for a description of the sample preparation, details of the phonon band structure obtained from the *ab initio* simulations, diffraction data refinement, hydrogen bond geometry, detailed results of the *ab initio* molecular dynamics simulations, and additional results of the neutron Compton scattering experiments.

## Data Availability

The data that support the findings of this study are openly available in Zenodo at https://doi.org/10.5281/zenodo.16954912, Ref. 99. Raw data were generated at ISIS Spallation Source, Rutherford Appleton Laboratory, UK. Derived data supporting the findings of this study are available from the corresponding author upon reasonable request.
